# Early assessment of circulating tumor DNA after curative‐intent resection predicts tumor recurrence in early‐stage and locally advanced non‐small‐cell lung cancer

**DOI:** 10.1002/1878-0261.13116

**Published:** 2021-10-31

**Authors:** Silvia Waldeck, Jan Mitschke, Sebastian Wiesemann, Michael Rassner, Geoffroy Andrieux, Max Deuter, Jurik Mutter, Anne‐Marie Lüchtenborg, Daniel Kottmann, Laurin Titze, Christoph Zeisel, Martina Jolic, Ulrike Philipp, Silke Lassmann, Peter Bronsert, Christine Greil, Justyna Rawluk, Heiko Becker, Lisa Isbell, Alexandra Müller, Soroush Doostkam, Bernward Passlick, Melanie Börries, Justus Duyster, Julius Wehrle, Florian Scherer, Nikolas von Bubnoff

**Affiliations:** ^1^ Department of Medicine I Medical Center – University of Freiburg Faculty of Medicine University of Freiburg Freiburg Germany; ^2^ German Cancer Consortium (DKTK) Partner Site Freiburg German Cancer Research Center (DKFZ) Heidelberg Germany; ^3^ Department of Thoracic Surgery Medical Center – University of Freiburg Faculty of Medicine University of Freiburg Freiburg Germany; ^4^ Institute of Medical Bioinformatics and Systems Medicine Medical Center – University of Freiburg Faculty of Medicine University of Freiburg Freiburg Germany; ^5^ Institute for Surgical Pathology Medical Center – University of Freiburg Faculty of Medicine University of Freiburg Freiburg Germany; ^6^ Institute for Neuropathology Medical Center – University of Freiburg Faculty of Medicine University of Freiburg Freiburg Germany; ^7^ Department of Hematology and Oncology University Hospital Schleswig‐Holstein Lübeck Germany

**Keywords:** circulating tumor DNA, early‐stage and locally advanced non‐small‐cell lung cancer, minimal residual disease, noninvasive biomarker, relapse prediction

## Abstract

Circulating tumor DNA (ctDNA) has demonstrated great potential as a noninvasive biomarker to assess minimal residual disease (MRD) and profile tumor genotypes in patients with non‐small‐cell lung cancer (NSCLC). However, little is known about its dynamics during and after tumor resection, or its potential for predicting clinical outcomes. Here, we applied a targeted‐capture high‐throughput sequencing approach to profile ctDNA at various disease milestones and assessed its predictive value in patients with early‐stage and locally advanced NSCLC. We prospectively enrolled 33 consecutive patients with stage IA to IIIB NSCLC undergoing curative‐intent tumor resection (median follow‐up: 26.2 months). From 21 patients, we serially collected 96 plasma samples before surgery, during surgery, 1–2 weeks postsurgery, and during follow‐up. Deep next‐generation sequencing using unique molecular identifiers was performed to identify and quantify tumor‐specific mutations in ctDNA. Twelve patients (57%) had detectable mutations in ctDNA before tumor resection. Both ctDNA detection rates and ctDNA concentrations were significantly higher in plasma obtained during surgery compared with presurgical specimens (57% versus 19% ctDNA detection rate, and 12.47 versus 6.64 ng·mL^−1^, respectively). Four patients (19%) remained ctDNA‐positive at 1–2 weeks after surgery, with all of them (100%) experiencing disease progression at later time points. In contrast, only 4 out of 12 ctDNA‐negative patients (33%) after surgery experienced relapse during follow‐up. Positive ctDNA in early postoperative plasma samples was associated with shorter progression‐free survival (*P* = 0.013) and overall survival (*P* = 0.004). Our findings suggest that, in early‐stage and locally advanced NSCLC, intraoperative plasma sampling results in high ctDNA detection rates and that ctDNA positivity early after resection identifies patients at risk for relapse.

## Graphical abstract

ctDNA profiling in plasma revealed higher levels and detection rates of ctDNA during tumor resection as compared to pretreatment time points in patients with early‐stage or locally advanced NSCLC. Moreover, patients testing positive for ctDNA immediately after tumor resection had worse clinical outcomes than patients with undetectable ctDNA. Our research highlights the role of ctDNA assessment for guiding treatment in patients with respectable NSCLC.

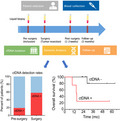

**Abbreviations**
AF: allele frequency
BWA: Burrows–Wheeler aligner
cfDNA: cell‐free DNA
ctDNA: circulating tumor DNA
FFPE: formalin‐fixed paraffin‐embedded
kb: kilobases
MRD: minimal residual disease
NGS: next‐generation sequencing
NSCLC: non‐small‐cell lung cancer
OS: overall survival
PCR: polymerase chain reaction
PDWB: plasma‐depleted whole blood
PFS: progression‐free survival
qPCR: quantitative polymerase chain reaction
RECIST: response evaluation criteria in solid tumors
SNP: single nucleotide polymorphism
SNV: single nucleotide variant

## 1 Introduction

Lung cancer is the leading cause of cancer‐associated death worldwide [1], with 80% of deaths being attributable to non‐small‐cell lung cancer (NSCLC) [2]. Patients with early‐stage and locally advanced NSCLC usually receive curative‐intent first‐line treatment such as tumor resection, radiotherapy, and/or combined approaches such as radiochemotherapy and adjuvant systemic treatment including chemotherapy or immune checkpoint blockade [3, 4, 5]. However, a large proportion of stage I–III NSCLC patients will experience disease progression, which is associated with a particularly poor prognosis [6]. Postoperative five‐year survival is 35%–55% for stage II and 15%–40% for stage III patients [7]. Thus, there is an unmet clinical need for accurate and robust biomarkers that identify patients at high risk for future NSCLC recurrence after curative‐intent therapies [8].

Profiling of circulating tumor DNA (ctDNA) in the blood plasma of cancer patients has shown great potential as a noninvasive biomarker for tumor genotyping and classification, monitoring of minimal residual disease (MRD), and detection of clonal heterogeneity over time [9, 10, 11, 12, 13]. A few studies have shown that ctDNA positivity after curative‐intent treatment may identify patients at higher risk for relapse [12, 13, 14, 15, 16]. However, defining the most robust and practical time point for ctDNA assessment after surgery remains a major challenge.

In general, pretreatment ctDNA detection rates in early‐stage and locally advanced NSCLC were substantially lower than in advanced metastasized stage IV lung cancer, mainly due to lower tumor sizes and minimal shedding of DNA molecules into the bloodstream [10, 17]. Manual manipulation of tumors during surgery potentially increases ctDNA release, suggesting that intraoperative blood collection might be a promising alternative for genetic profiling of ctDNA in NSCLC patients undergoing surgical tumor resection [18, 19, 20].

Here, we present the results of a prospective study in resectable stage I‐III NSCLC. We profiled ctDNA before, during, and after tumor resection to evaluate the impact of intraoperative procedures on ctDNA characteristics and to assess its value as a biomarker for early outcome prediction.

## 2 Methods

### 2.1 Study design and patients

Patients with resectable early‐stage or locally advanced NSCLC (stage I–III) qualified for inclusion into this trial (DRKS00009521). From October 2014 to June 2018, a total of 33 patients aged ≥ 18 years were enrolled at the University Medical Center Freiburg, Germany. From 21 patients, both tumor biopsies and serial blood plasma samples were available for further analyses (Fig. S1 and Table S1). Additionally, blood from six healthy individuals was collected to define the tumor genotyping threshold for ctDNA monitoring and to assess the rate of false‐positive ctDNA detection (see below). All patients and healthy controls gave written informed consent. This study (DRKS00009521) was conducted in accordance with the Declaration of Helsinki and was approved by the local Institutional Review Board and Ethics committee (Nr. 126/14, Date 09/23/2014). Patients were treated according to international standards and local guidelines.

### 2.2 Sample collection and processing

**Fig. 1:**
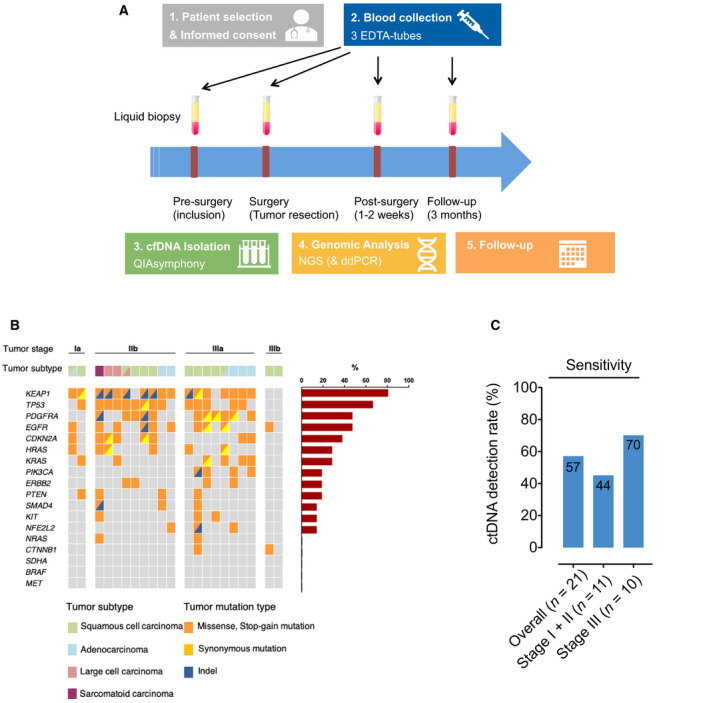
Overview of the study design, detected tumor mutations of patients, and sensitivity of ctDNA analysis by our custom NGS panel. (A) Overview of the study design with standardized time points of blood plasma collection. (B) Overview of clinicopathological features and tumor mutations. Each column represents the data from one patient (left). Bar chart showing the frequency of detected mutations in the respective gene (right). (C) Bars showing sensitivity of ctDNA detection in pretreatment plasma samples by our custom molecular barcode‐containing capture strategy. cfDNA, circulating tumor DNA; NGS, next‐generation sequencing; ddPCR, digital‐droplet polymerase chain reaction; Indel, insertion/deletion.

Blood samples from 21 NSCLC patients were collected presurgery at enrollment, during surgery from the jugular vein through a central venous catheter immediately after pulmonary vein clamping and before tumor resection (after manual palpation of the lung and tumor lesions), 1–2 weeks after surgery (end of hospitalization, in median 10 days after surgery), and during follow‐up every 3 months (Fig. 1). At each time point, 2 EDTA samples with a total of 18 mL blood (2 x 9 mL) were collected and processed within 2 h after collection. At presurgery and follow‐up time points, blood was collected from peripheral veins, during surgery from the jugular vein. Blood from healthy individuals was processed in the same fashion.

Blood plasma was separated from the cellular blood compartment by centrifugation at 800 ***g*** for 10 min at room temperature. The plasma compartment was then centrifuged again at 1000 ***g*** for 10 min and subsequently stored at −80 °C. Likewise, plasma‐depleted whole blood (PDWB) was stored at −80 °C.

Cell‐free DNA (cfDNA)‐isolation from blood plasma was performed using the QIAsymphony and the QIAmp Circulating Nucleic Acid Kit (Qiagen, Hilden, Germany) according to the manufacturer´s instructions. Quality and quantity of the isolated cfDNA were assessed by Fragment Analyzer (Agilent Technologies, Santa Clara, CA, USA) and Qubit 2.0 fluorometer (Thermo Fisher Scientific, Waltham, MA, USA). Isolated cfDNA was then stored at −20 °C.

DNA from primary tumor resection specimens (*n* = 21) was isolated from formalin‐fixed paraffin‐embedded (FFPE) tissue specimens after microdissection of the tumor areas using the GeneRead DNA FFPE Kit (Qiagen, Hilden, Germany). DNA quality and quantity of these samples were assessed as described above.

Cellular DNA from PDWB was used as germline control to identify single nucleotide polymorphisms (SNP) in all patients. To isolate cellular DNA from PDWB, cells were lysed by the addition of an NH_4_Cl solution (8.4 g NH_4_Cl soluted in 1 L H2O) and incubated for 20 min at room temperature, followed by one centrifugation step for 10 min at 800 ***g***. Lysed cells were then washed twice with PBS and lysis was completed by the addition of RLT+‐buffer (Qiagen, Hilden, Germany) and homogenization with a syringe. Cell lysates were stored at −80 °C until further use. Cellular DNA from the lysates was isolated using the QIAsymphony and the ‘QIASymphony DSP DNA Mini Kit 192 version 1’ according to the manufacturer`s protocol and stored at −20 °C. In cases with no available PDWB, cellular DNA for SNP identification was obtained from segments of FFPE tissues with no evidence of histological tumor infiltration. Cellular DNA from these parts was isolated as described above, using the GeneRead DNA FFPE Kit (Qiagen, Hilden, Germany).

### 2.3 Next‐generation sequencing workflow

Cellular DNA (i.e., DNA from tumor biopsies and PDWB) was sheared prior to library preparation by Covaris (Covaris, Woburn, MA, USA), using the following settings: 180 s with a duty factor of 10%, a peak incident power (W) of 175, and 200 cycles/burst. Next‐generation sequencing (NGS) libraries were generated from both cellular sheared DNA and cfDNA with the ThruPlex Tag‐Seq 48S Kit (Takara, Kusatsu, Japan), using molecular barcodes (unique identifiers, UID) according to the manufacturer's instructions. In median, 17.2 ng (range: 6.2–59.4 ng) of cellular tumor DNA, 50 ng (range: 1.74–50 ng) of cellular DNA from PDWB, and 13.2 ng (range: 0.6–90 ng) of cfDNA were used for library preparation. After generating NGS libraries, target enrichment with complementary probes was performed using a customized targeted‐capture panel (Integrated DNA Technologies, Coralville, IA, USA), according to the manufacturer's instructions. The following 18 commonly mutated genes in NSCLC were selected for the customized sequencing panel (17 kb), based on the COSMIC database (https://cancer.sanger.ac.uk/cosmic): *BRAF* (exons 11, 15), *CDKN2A* (exons 1, 2), *CTNNB1* (exon 3), *EGFR* (exons 18, 19, 20, 21), *HER2/ERBB* (exon 20), *HRAS* (exons 2, 3), *KEAP1* (exons 2, 3, 4, 5, 6), *KIT* (9, 11, 13, 14, 17), *KRAS* (2, 3, 4), *MET* (exon 14), *NFE2L2* (exon 2), *NRAS* (exons 2, 3), *PDGFRA* (exon 18), *PIK3CA* (exons 10, 21), *PTEN* (exons 1, 3, 6, 7, 8), *SDHA* (exons 2, 7, 13), *SMAD4* (exons 1, 2, 8, 9, 10, 11), *TP53* (exons 4, 5, 6, 7, 8, 9, 10) (Table S2). Quantification and quality assessment of the post‐capture libraries and the multiplex‐NGS pool were performed by Fragment Analyzer (Agilent Technologies, Santa Clara, CA, USA), Qubit 2.0 fluorometer (Thermo Fisher Scientific, Waltham, MA, USA), and by qPCR (LightCycler Rotor‐Gene Q, Qiagen, Hilden, Germany), using the KAPA Library Quantification Kit (Roche, Basel, Switzerland). Paired‐end sequencing of the multiplex‐NGS pool (2 x 125 bp) was performed by the ‘Genomics and Proteomics Core Facility’ of the DKFZ Heidelberg (Germany) on a HiSeq 2000 (Illumina, San Diego, CA, USA).

### 2.4 Sequence data analysis, tumor genotyping, and ctDNA monitoring

Paired‐end reads were mapped to the hg19 reference genome (GRCh37v28/hg19) with Burrows–Wheeler aligner (BWA) version 0.7.17 (default parameters), allowing soft‐clipping, and sorted and indexed with SAMtools. Deduplication was performed using Connor (https://github.com/umich‐brcf‐bioinf/Connor), applying a consensus frequency threshold of 90% and a family size threshold of 10. Median deduplication rate was 91%. Variant calling of single nucleotide variants (SNVs) was performed using the software Sequence Pilot (JSI medical systems GmbH, Ettenheim, Germany). SNPs were identified by sequencing each patient’s cellular DNA and discarded for further analyses.

A threshold of ≥ 18% allele frequency (AF) was defined for tumor genotyping based on the false‐positive rate in plasma samples from healthy controls (see below). SNVs passing this threshold were used for ctDNA monitoring analyses in blood plasma. Here, plasmas samples were considered ctDNA‐positive if the following rules applied: (a) detection of at least one predefined SNV from tumor genotyping; (b) a mean AF of at least 0.001% for the plasma sample (see ‘Statistics’ for definition of ‘mean AF’). This detection limit of 0.001% was established as follows: First, spike‐in experiments using artificial mutant DNA (Horizon cfDNA reference standards, USA) revealed robust detection of SNVs above an AF of 0.1% (Fig. S2A). We then used this threshold to assess the false‐positive rate and the AF detection limit. Here, each patient’s SNV from tumor genotyping was monitored in cfDNA from six healthy donors (*n* = 126 tests). At a threshold of 0.001% mean AF and a threshold of ≥ 18% for tumor genotyping, a false‐positive rate of 5% was observed (Fig. S2B).

### 2.5 Statistics

Mutant allele frequencies were expressed as percentage (%) and set to the number of reads harboring a given variant divided by the total number of reads at the same genomic position. The AF of total ctDNA was calculated as the mean AFs of all tumor‐derived mutations detected by monitoring in blood plasma.

Further statistical analyses were performed using graphpad version 8.0 software (GraphPad Software, San Diego, CA, USA). To compare continuous data distributions, we used the nonparametric Mann–Whitney *U*‐test. Fisher’s exact test was applied to compare proportions of two nominal variables. For Kaplan–Meier analyses, log‐rank tests were used to statistically evaluate survival differences between two groups. For clinical outcome analyses, progression‐free survival (PFS) was considered the primary endpoint, defined as time from blood sample collection to any of the following: disease progression as defined by RECIST 1.1‐based radiographic assessment, death from any cause or last follow‐up visit. Overall survival (OS) was considered as secondary endpoint and was defined as the time from blood sample collection to death from any cause or last follow‐up. *P*‐values < 0.05 were considered as significant.

## 3 Results

We enrolled a total of 33 stage I‐III NSCLC patients in our study. From 21 patients, tumor biopsies as well as presurgical plasma and serial plasma samples were available for both tumor genotyping and ctDNA analyses (Fig. S1). Patient characteristics are shown in Tables 1 and S1. Most patients were diagnosed with locally advanced disease stage IIB to IIIB (*n* = 18/21, 85.7%). The two most frequent histopathology subtypes were squamous cell carcinoma (47.6%) and adenocarcinoma (23.8%). All patients underwent curative‐intent tumor resection that was followed by either adjuvant chemotherapy (*n* = 9), radiotherapy (*n* = 2), combined chemoradiation (*n* = 2), or no adjuvant treatment (*n* = 10, Table S1). We applied our customized targeted‐capture high‐throughput sequencing approach to a total of 21 primary FFPE tumor specimens and 96 plasma samples obtained at enrollment (presurgery), during tumor resection, early after surgery (after 1–2 weeks, at the end of hospitalization), and every 3 months after surgery during follow‐up (Fig. 1A, Table S1 and Table S3). Median follow‐up was 26.2 months.

**Table 1:** Patient characteristics.

Characteristic	No. of patients (*n*, %)
Age	
Median (in years)	70
Range (in years)	48‐85
Sex	
Female	7 (33%)
Male	14 (67%)
Smoking history	
Positive	21 (100%)
Negative	0 (%)
T‐Stage	
T1	3 (14.3%)
T2	7 (33.3%)
T3	7 (33.3%)
T4	4 (19.1%)
N‐Stage	
N0	6 (28.6%)
N1	10 (47.6%)
N2	4 (19.0%)
N3	1 (4.8%)
M‐Stage	
M0	21 (100%)
M1	0 (0%)
Tumor stage	
IA	2 (9.5%)
IB	0 (0%)
IIA	1 (4.8%)
IIB	8 (38.1%)
IIIA	8 (38.1%)
IIIB	2 (9.5%)
Resection status	
R0	19 (90.5%)
R1	2 (9.5%)
Histological Subtype	
Adenocarcinoma	5 (23.8%)
Squamous cell carcinoma	10 (47.6%)
Sarcomatoid carcinoma	1 (4.8%)
Large cell carcinoma	2 (9.5%)
Other	3 (14.3%)
Therapy	
Tumor resection	21 (100%)
Adjuvant chemotherapy	9 (42.8%)
Adjuvant radiotherapy	2 (9.5%)
Adjuvant radiochemotherapy	2 (9.5%)

Tumor tissue genotyping revealed a median of 10 mutations per patient, with *KEAP1* being the most affected gene (81% of patients), followed by *TP53* (67%), *EGFR,* and *PDGFRA* (52%, Fig. 1B, Table S4). Other mutations were detected in *KRAS*, *HRAS*, *PIK3CA*, *ERBB2*, *PTEN*, *SMAD4*, *KIT*, *NFE2L2*, *CDKN2A*, *NRAS,* and *CTNNB1* genes (Fig. 1B, Table S4). Using the identical platform, we next analyzed plasma samples collected at pretreatment time points and during surgery to determine sensitivity for detection of tumor mutations in ctDNA. Plasma samples from six healthy individuals were analyzed to assess the false‐positive rate of our sequencing approach and to define the tumor genotyping AF threshold (see Methods). Overall, we identified tumor‐specific mutations in ctDNA with a sensitivity of 57% (12 out of 21 patients) before tumor resection (i.e., either presurgery or during surgery) (Fig. 1C), with stage III NSCLC patients revealing higher ctDNA detection rates (70%) than stage I–II patients (44%, Fig. 1C).

**Fig. 2:**
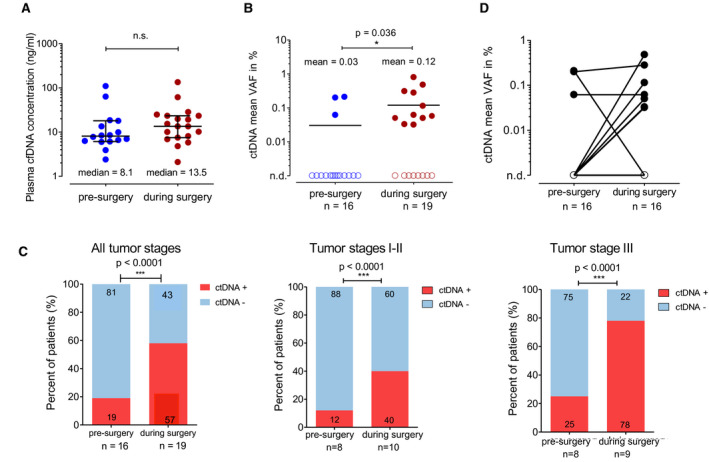
ctDNA in presurgical and intrasurgical plasma samples (A) Comparison of cfDNA concentrations isolated from patient’s plasma presurgery and during surgery. Lines represent the median and interquartile range. (B) Percentage of patients with positive or negative ctDNA status presurgery and during surgery. Lines represent the mean. (A, B) Mann–Whitney *U*‐test was used to compare the two groups. (C) Comparison of ctDNA detection rates between plasma samples obtained presurgery or during surgery in all patients, stage I–II, and stage III patients. Fisher’s exact test was performed to assess statistical differences between the two groups. (D) ctDNA concentrations of paired plasma samples from the same patients, connected through a black line. n.d., not detected; cfDNA, cell‐free DNA; ctDNA, circulating tumor DNA.

We then assessed and compared detection rates in patient’s plasma samples collected before surgery (*n* = 16) with those obtained during surgery after manual lung and tumor palpation and immediately before tumor resection (*n* = 19). Samples collected during surgery had higher cfDNA concentrations (median of 13.5 ng·mL^−1^ versus 8.1 ng·mL^−1^, n.s., Fig. 2A) and significantly higher ctDNA levels compared to preinterventional specimens (mean of 0.12% AF versus 0.03% AF, *P* = 0.036, Fig. 2B). Consequently, intraoperative samples yielded higher ctDNA detection rates across disease stages (Figs 2C and S3). Comparing pretreatment samples with matched plasma samples obtained during tumor resection in individual cases, we observed an increase of ctDNA in the vast majority of patients (Fig. 2D). Finally, patients with positive ctDNA during surgery showed a nonsignificant trend for unfavorable PFS and OS compared to patients with negative ctDNA (Fig. S4).

**Fig. 3:**
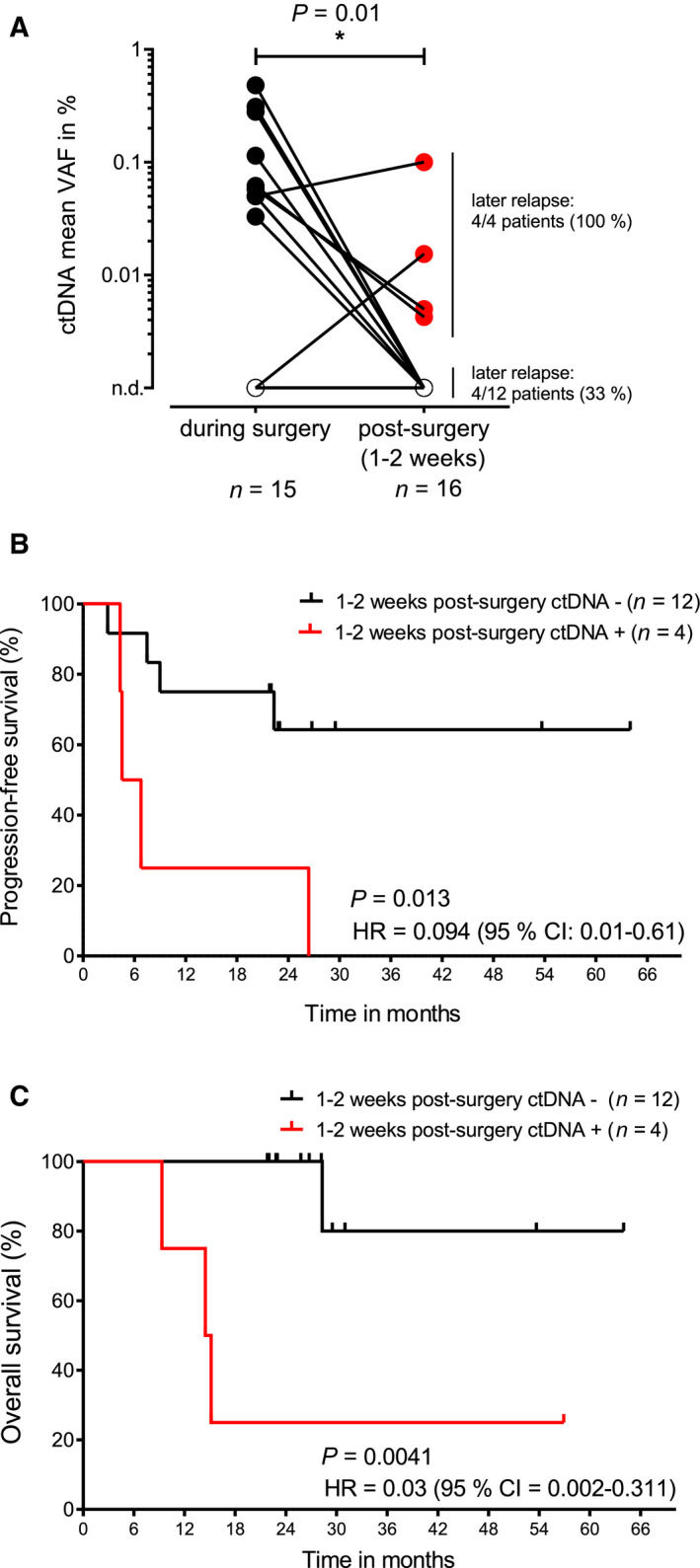
The value of ctDNA for outcome prediction early after surgery. (A) Comparison of mean ctDNA VAFs during surgery and after tumor resection (1–2 weeks postsurgery). Paired values from the same patients are connected through a line. Mann–Whitney *U*‐test was used to assess differences between the two groups. (B) PFS in patients with positive and negative ctDNA status 1‐2 weeks after surgery. (C) Overall survival in patients with positive and negative ctDNA status 1–2 weeks after surgery. In (B and C) log‐rank tests were used to assess significance. n.d., not detected; HR, hazard ratio; ctDNA, circulating tumor DNA; VAF, variant allele frequency.

In a next step, we evaluated whether the presence of ctDNA as MRD early after tumor resection could predict relapse and patient clinical outcomes. We applied our NGS method to 16 plasma samples uniformly collected 1–2 weeks after surgery to monitor previously defined tumor‐derived SNVs in cfDNA (Fig. 3). We found 12 patients with no evidence of ctDNA and 4 patients with detectable MRD early after tumor resection. While 100% of ctDNA‐positive patients (4/4) experienced later disease relapse (mean lead time: 10.31 months), 67% of those with a negative result remained disease‐free during follow‐up (Fig. 3A). Relapse tumors in the 4 patients with negative ctDNA postoperatively were located in the brain (*n* = 2), contralateral lung (*n* = 1), and liver (*n* = 1). Furthermore, MRD negativity was significantly associated with both favorable PFS (*P* = 0.013, 1‐year PFS: 25% versus 75%, HR 0.094 [95% CI 0.01–0.061], Fig. 3B) and OS (*P* = 0.004, 2‐year OS: 25% versus 80%, HR 0.03 [95% CI 0.002–0.311], Fig. 3C), highlighting the value of ctDNA after surgery as an early prognostic biomarker in stage I‐III NSCLC.

**Fig. 4:**
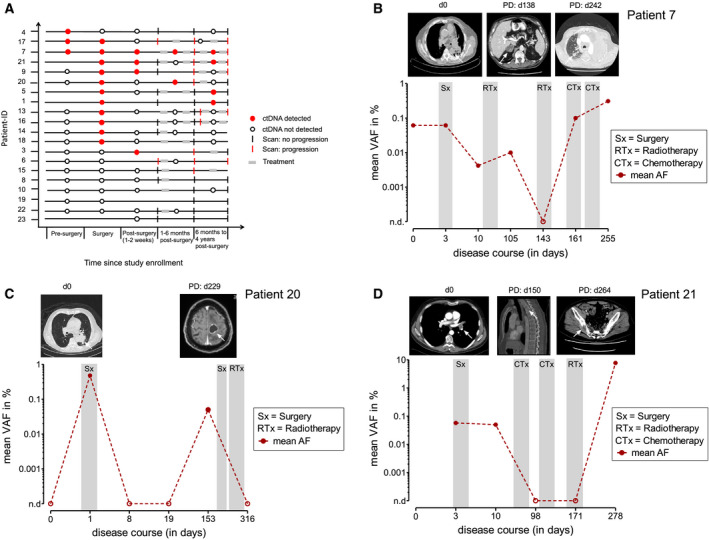
ctDNA as biomarker for disease surveillance. (A) Event chart showing the course of the disease with results of radiographic assessment and ctDNA analyses for each patient. Red circle, ctDNA detected; empty circle; ctDNA not detected; black line, imaging studies showing complete response; red lines, imaging studies show detection of disease. (B–D) Change of disease burden in response to treatment and during clinical progression in three different patients. Shown is the mean AF of all monitored SNVs in plasma over serial time points. ctDNA, circulating tumor DNA; Sx, Surgery; RTx, radiotherapy; CTx, chemotherapy; PD, progressive disease; VAF, variant allele frequency.

Finally, we profiled plasma up to 4 years after tumor resection to monitor the disease over time (Fig. 4A). In five cases, relapse or progression was preceded by negative to positive conversion of ctDNA or an increase of ctDNA concentrations. In four cases, ctDNA was never positive during follow‐up despite radiological evidence of disease relapse. Cases 1 and 5 had detectable mutations in *TP53* (V272L) and *KEAP1* (S338STOP) in one single plasma sample during follow‐up without any signs of radiological disease progression, indicating false‐positive ctDNA identification (Fig. 4A).

In general, ctDNA concentrations robustly mirrored tumor burden over time in our patient cohort. For example, patient 7 experienced multiple disease progressions reflected by increasing ctDNA concentrations after an initial drop following tumor resection (Fig. 4B). Patient 20 was ctDNA‐positive in plasma samples obtained during tumor resection and turned negative 8 days after surgery. Five months later, the identification of tumor‐specific mutations in the blood was followed by the occurrence of brain metastases, predicting CNS relapse 76 days before radiographic detection (Fig. 4C). Patient 21 was diagnosed with combined small‐cell and non‐small‐cell squamous lung carcinoma. Here, ctDNA remained positive early after surgery, predicting radiographic disease relapse 5 months later. After combined radiochemotherapy, the patient again experienced disease progression with multiple bone metastases, reflected by an increase of ctDNA up to an AF of 10% (Fig. 4D).

## 4 Discussion

The prognosis of stage I‐III NSCLC patients experiencing disease relapse after curative‐intent therapy is poor. Accurate biomarkers to identify patients at high risk for NSCLC recurrence are lacking. To address these challenges, we applied a custom‐targeted‐capture high‐throughput sequencing approach to prospectively collected tumor biopsies (*n* = 21) and plasma samples (*n* = 96) from major disease landmarks to characterize the role of ctDNA as a noninvasive biomarker in this patient population. We demonstrate that ctDNA can be identified in 57% of patients, with higher detection rates in locally advanced stage III compared to early‐stage NSCLC (stage I‐II), in accordance with previous studies [10, 12, 13, 15, 17].

One major finding of our study was that ctDNA concentrations and detection rates significantly increased in intraoperative blood samples compared to blood obtained at presurgical time points. Those blood samples were collected in a standardized fashion during surgery from the jugular vein after pulmonary vein clamping and immediately before tumor resection. At this stage, lung tissue and tumor masses had been manually palpated by the surgeons, indicating that physical manipulation might contribute to shedding of ctDNA into the bloodstream. This effect seems to outweigh the impact of general surgical trauma, which has been shown to substantially hamper the detection of ctDNA through an increase of the general cfDNA pool and dilution of ctDNA molecules [21, 22, 23]. Indeed, while cfDNA levels increased by almost twofold during surgery in our study, ctDNA concentrations were found to be four times higher, supporting our assumption that manual manipulation of the tumor might play an important role for ctDNA shedding. An alternative hypothesis for improved ctDNA detection during surgery might be the proximity of blood draws to the tumor location. Previous studies have demonstrated that blood obtained from pulmonary veins harbored higher amounts of circulating tumor cells than blood drawn from peripheral veins [24, 25, 26].

Our second major finding was that ctDNA positivity 1–2 weeks after tumor resection potentially identified patients with a particularly high risk of future NSCLC relapse. While 100% of patients with detectable MRD early after surgery experienced disease progression (*n* = 4), almost two‐thirds of patients with negative MRD remained cancer‐free. Despite the rather small sample size of our study, these results, together with similar findings from other previously reported studies, suggest that ctDNA could potentially serve as a specific marker for relapse prediction and thus could guide adjuvant treatment decisions in the postoperative setting [8, 12, 14, 16, 27]. As Abbosh *et al*. [27] recently pointed out, thousands of patients and a long follow‐up would be required to prove an effect of adjuvant treatment after tumor resection in early‐stage NSCLC. Therefore, monitoring of ctDNA potentially offers a more sensitive and specific approach to identify those patients who would most likely relapse and thus might benefit from adjuvant therapies, and could lead to a new generation of adjuvant clinical trials. On the other hand, it would also spare patients without detectable ctDNA from toxicity of adjuvant therapies [27].

The median time from surgery to discharge from hospital was 10 days in our study. The first postoperative blood draw was performed at the end of hospitalization, that is, 1–2 weeks after surgery. Previous publications demonstrate rapid clearance of ctDNA after tumor resection and suggest a rather immediate postoperative blood draw (e.g., 3 days postsurgery) for MRD assessment [14]. However, this approach bears the risk of false‐positive results due to the persistence of ctDNA early after tumor resection, leading to potential overtreatment in the adjuvant setting [14]. Our findings, together with two other recently published studies, demonstrate that a blood draw at the end of hospitalization is both practical (because patient has not been discharged yet) and allows sensitive MRD detection and identification of patients at risk for disease progression [8, 16].

However, our study harbors some limitations. First, the sample size of our prospective cohort is rather small and larger studies are needed to validate the findings of this work. Furthermore, sensitivity of our sequencing approach could be increased by an alternative panel design. Our panel captures 18 NSCLC‐specific genomic regions and covers 17 kb of the genome. Yet, it has been shown that tracking a larger number of mutations per patient improves detection rates in solid cancers, especially in NSCLC [17, 27, 28]. This can be achieved in two ways: (a) by adding more genomic regions to ‘off‐the‐shelf’ panels that capture a higher number of entity‐specific variants, as shown in the work from Newman *et al*. (125 kb and 203 kb panels) [17, 28] and (b) by tracking a set of personalized genetic aberrations that have been identified through comprehensive sequencing of the tumor [10, 29, 30].

## 5 Conclusions

In conclusion, our work highlights the advantages of ctDNA as a noninvasive biomarker in early‐stage and locally advanced NSCLC. Our findings suggest that intraoperative manual manipulation of the tumor leads to an increase of ctDNA in the blood and an increase of ctDNA detection rates. We further show that ctDNA detection early after tumor resection identifies patients at risk for relapse, indicating a potential future role as MRD marker for guiding adjuvant systemic treatment.

## Conflict of interest

F.S. received research funding from Roche Sequencing Solutions. S.L. received Advisory Board and/or scientific meeting/presentation sponsorship; Agilent, AstraZeneca, Illumina, Novartis, Roche, as well as research project sponsorship Bristol–Myers–Squibb. All other authors declare no conflict of interest.

## Author contributions

NvB, FS, and SWa involved in conceptualization; SWa, JaMi, JuMu, HB, JW, FS, and NvB involved in methodology; JaMi, SWa, GA, and MB involved in software preparation; SWa validated the data; SWa, JaMi, MR, GA, MD, MJ, UP, JW, and FS involved in formal analysis; SWa involved in investigation; SWi, A‐ML, DK, SS, LT, CZ, SL, PB, CG, JR, HB, LI, AM, SD, DP, BP, and JD contributed to resources; SWa and JM contributed to data curation; SWa, FS, and NvB. contributed to writing—original draft preparation; all authors contributed to writing—review and editing; SWa contributed to visualization; FS and NvB contributed to supervision; SWa contributed to project administration; FS and NvB acquired funding. All authors have read and agreed to the published version of the manuscript.

## Supporting information


**Fig. S1.** Flow chart of the prospective study with reasons for exclusion.
**Fig. S2.** Spike‐in and specificity analyses.
**Fig. S3.** ctDNA concentrations during surgery in stage I/II versus stage III NSCLC patients.
**Fig. S4.** ctDNA as a biomarker during treatment.

**Table S1.** Patient characteristics shown for all individual patients of the cohort.
**Table S2.** Target regions of the custom target capture NGS panel.
**Table S3.** Overview of total cfDNA amount subjected to custom target capture NGS approach as well as the ctDNA status of the individual patients at all analyzed time points presurgery, during surgery, postsurgery, and follow‐up.
**Table S4.** Overview of mutations detected by tumor genotyping.

## Data Availability

The data presented in this study are available on request from the corresponding authors. The data are not publicly available due to ethical restrictions.
