# Supplementary figures and images for: Early assessment of circulating tumor DNA after curative‐intent resection predicts tumor recurrence in early‐stage and locally advanced non‐small‐cell lung cancer
Source: Mol Oncol. 2021 Oct 31;16(2):527–37. doi: 10.1002/1878-0261.13116 (PMC8763652; doi:10.1002/1878-0261.13116)

Supplementary Figure 1

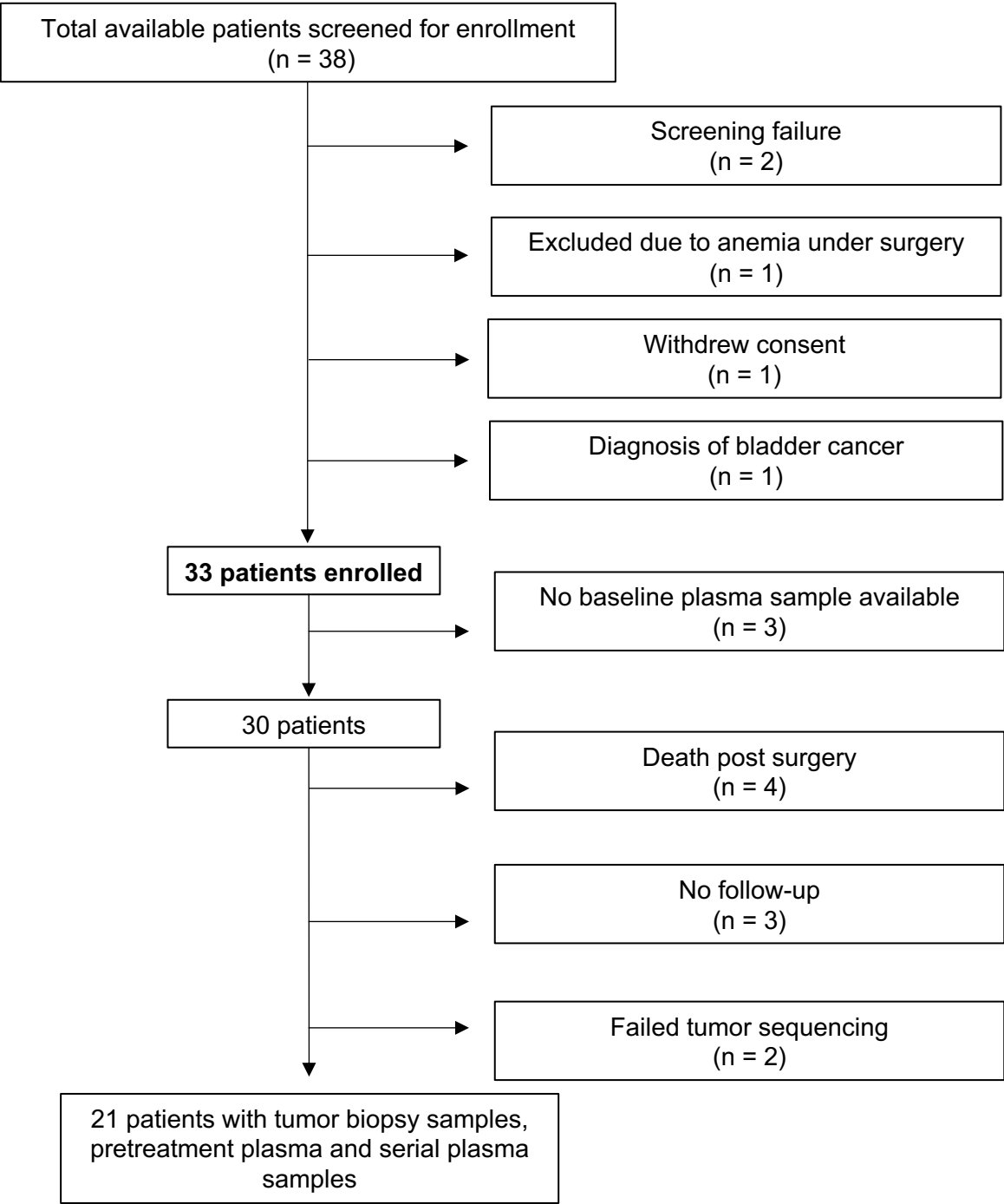

Supplementary Figure 2

**A**

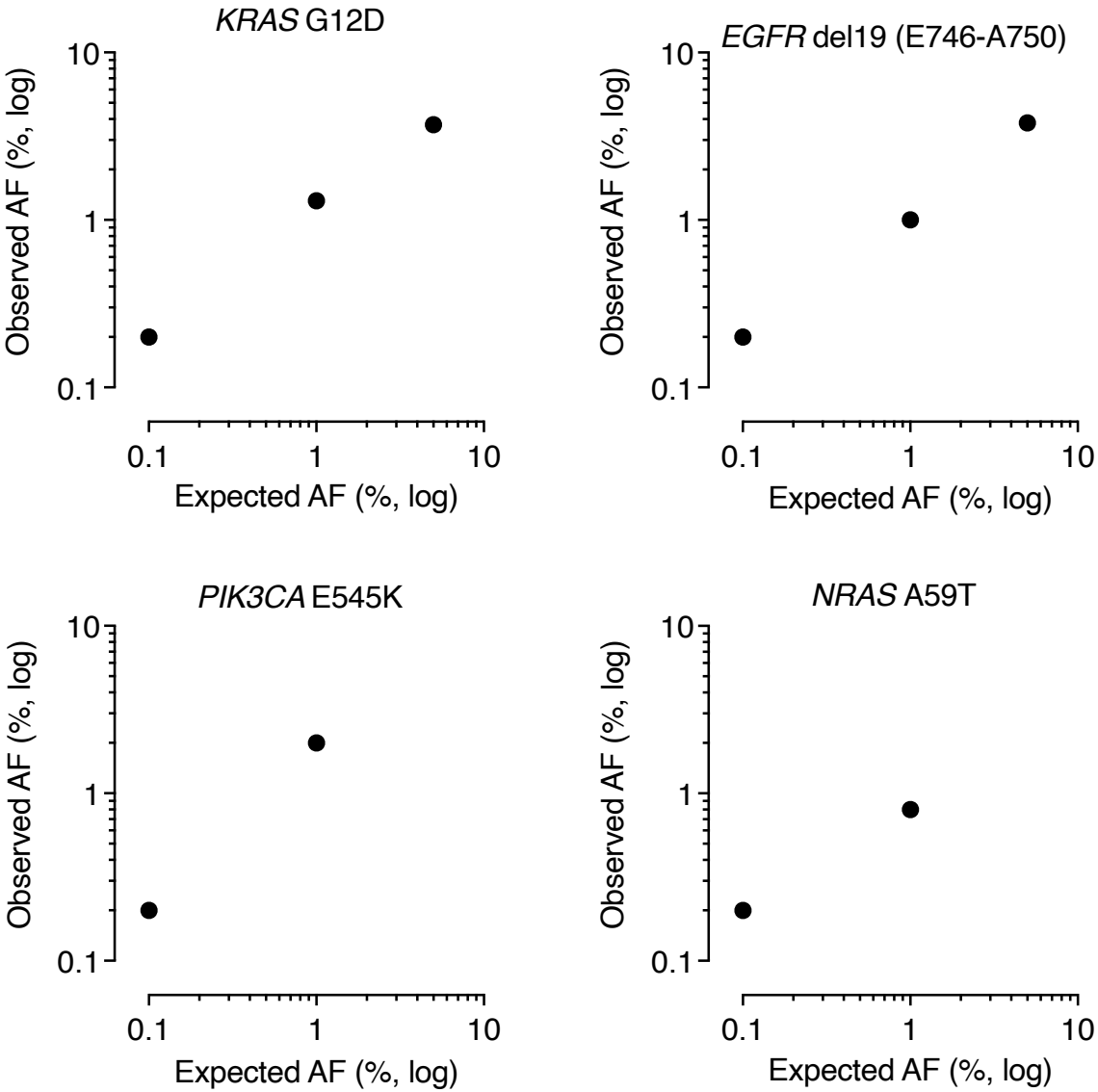

**B**

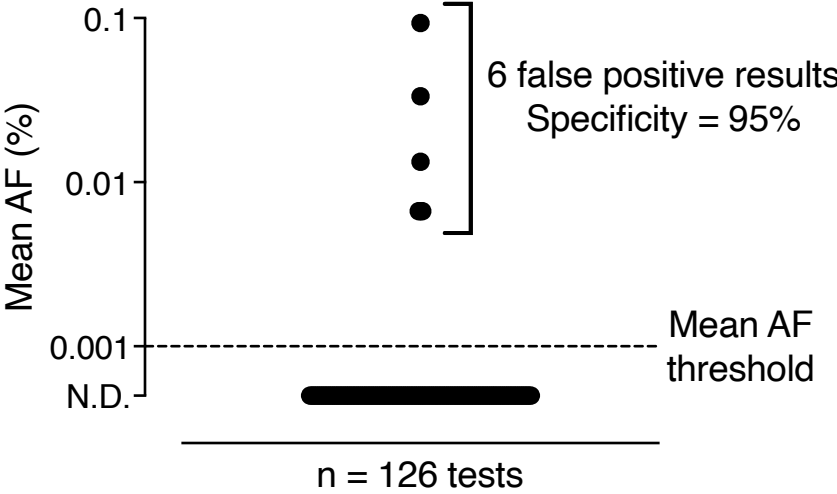

Supplementary Figure 3

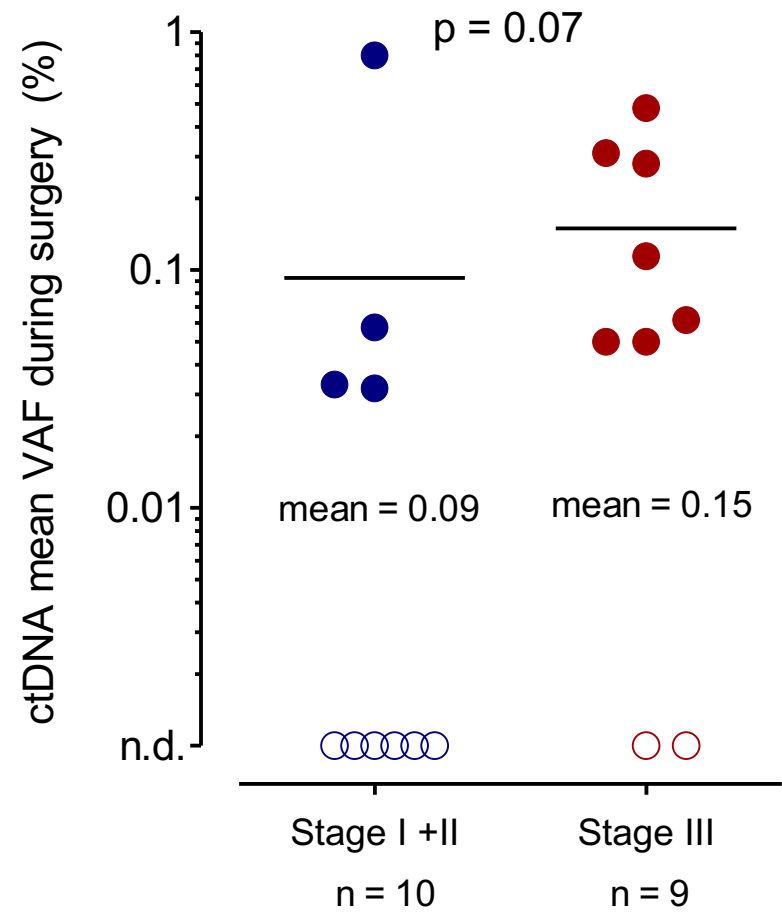

Supplementary Figure 4

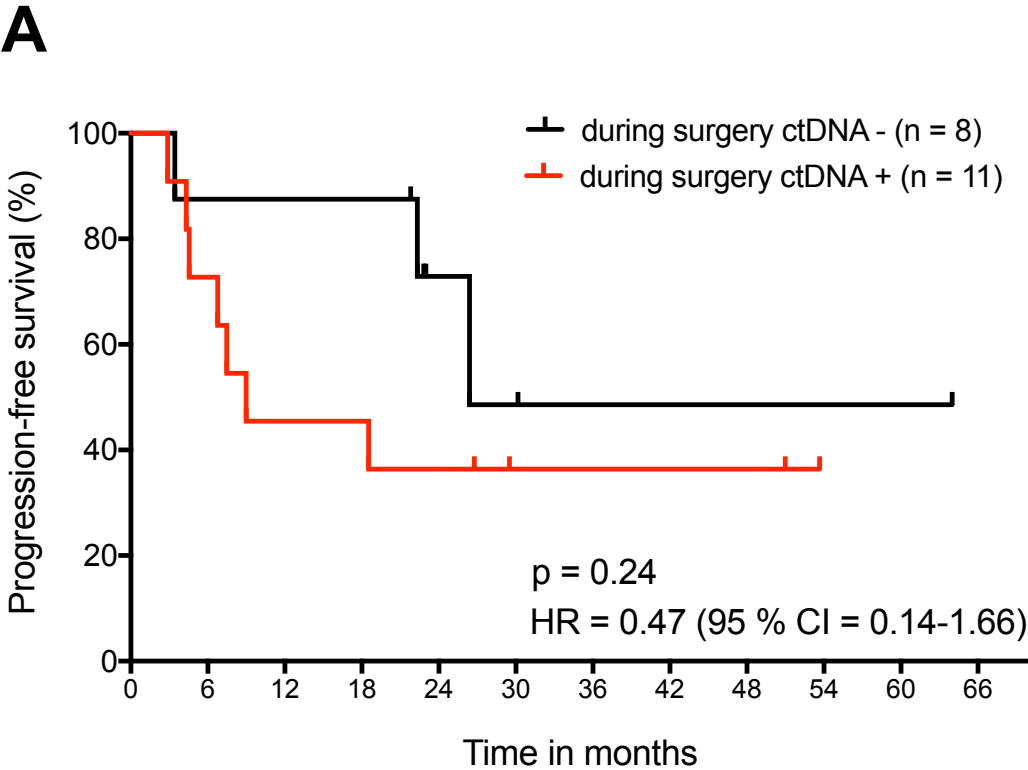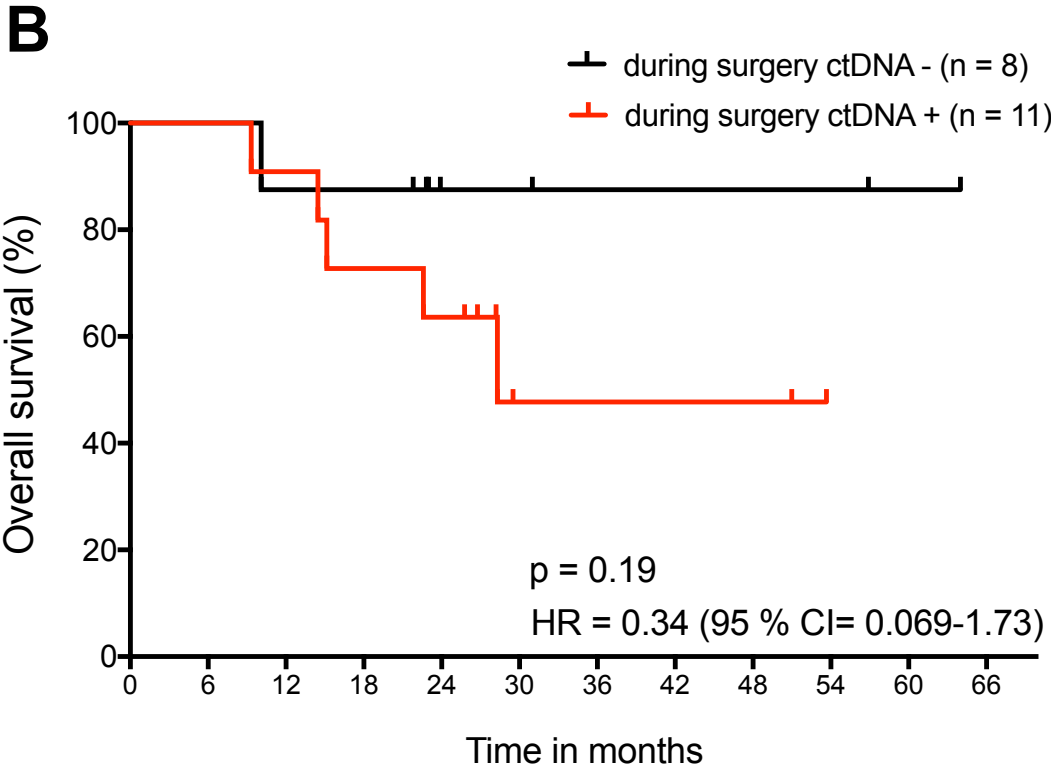

Supplement: Supplementary file 1 — Fig. S1. Flow chart of the prospective study with reasons for exclusion. Fig. S2. Spike‐in and specificity analyses. Fig. S3. ctDNA concentrations during surgery in stage I/II versus stage III NSCLC patients. Fig. S4. ctDNA as a biomarker during treatment. [file MOL2-16-527-s001.pdf]
